# Supplementary material for: Psychometric Properties of the Maternal and Paternal Parenting Styles Scale in Chilean Adolescents
Source: Int J Environ Res Public Health. 2021 Jun 9;18(12):6229. doi: 10.3390/ijerph18126229 (PMC8296027; doi:10.3390/ijerph18126229)
Supplement: Supplementary file 1 [file ijerph-18-06229-s001.zip › ijerph-1249253-supplementary.pdf]

ESCALA DE ESTILOS EDUCATIVOS MATERNO Y PATERNO

Puntúa en una escala del 1 al 6 cómo son de importantes para ti las siguientes cuestiones					
Totalmente en desacuerdo<br>1	En desacuerdo<br>2	Parcialmente en desacuerdo<br>3	Parcialmente de acuerdo<br>4	De acuerdo<br>5	Totalmente de acuerdo<br>6

**1**	Disfruta hablando cosas conmigo
**2**	Con frecuencia pasa algún tiempo hablando conmigo
**3**	Cuando hablo con él/ella muestra interés y atención
**4**	Me siento apoyado y comprendido por él/ella
**5**	Me anima a que le cuente mis problemas y preocupaciones
**6**	Me hace sentir mejor cuando estoy desanimado/a
**7**	Si tengo algún problema puedo contar con su ayuda
**8**	Muestra interés por mí cuando estoy triste y enfadado/a
**9**	Me anima a que le diga lo que pienso aunque él/ella no esté de acuerdo
**10**	Me anima a que exprese mis ideas aunque no gusten a otras personas
**11**	Piensa que aunque aún no sea una persona adulta puedo tener ideas acertadas
**12**	Me anima a que tome mis propias decisiones
**13**	Me anima que piense de forma independiente
**14**	Me permite opinar cuando hay que tomar una decisión familiar
**15**	Cuando quiere que haga algo me explica por qué quiere que lo haga
**16**	Me dice que siempre hay que mirar las dos caras de un problema
**17**	Intenta saber a dónde voy cuando salgo
**18**	Si vuelvo tarde a casa me pregunta por qué y con quién estuve
**19**	Cuando salgo un sábado noche debo decirle antes dónde voy y cuándo volveré
**20**	Intenta saber qué hago en mi tiempo libre
**21**	Pone límites a la hora a la que debo volver a casa
**22**	Me pregunta en qué gasto el dinero
**23**	Es menos amable conmigo cuando no hago las cosas a su manera
**24**	Siempre me está diciendo lo que tengo que hacer
**25**	Me hace sentir culpable cuando no hago lo que quiere
**26**	Me trata de forma fría y distante si hago algo que no le gusta
**27**	Me dice que él/ella tiene razón y no debo llevarla la contraria
**28**	Me castiga y sanciona sin darme explicaciones
**29**	Intenta controlar continuamente mi forma de ser y pensar
**30**	Deja de hablarme cuando se enfada conmigo
**31**	Le cuento lo que hago en mi tiempo libre
**32**	Le cuento lo que hago cuando salgo
**33**	Le hablo sobre los problemas que tengo con mis amigos y amigas
**34**	Cuando llego de la escuela le cuento cómo me ha ido el día
**35**	Aunque no me pregunte, le cuento cómo me va en las diferentes asignaturas
**36**	Suele estar de buen humor
**37**	Casi siempre es una persona alegre y optimista
**38**	Suele bromear conmigo
**39**	Es divertido hacer cosas con él/ella
**40**	Se ríe mucho conmigo

**41**	Suele estar tranquilo/a y relajado/a
